# Draft Genome Sequence of Achromobacter spanius Strain 6, a Soil Bacterium Isolated from a Hydrocarbon-Degrading Microcosm

**DOI:** 10.1128/MRA.01124-18

**Published:** 2018-09-20

**Authors:** Thusitha S. Gunasekera, Osman Radwan, Loryn L. Bowen, Lisa M. Brown, Oscar N. Ruiz

**Affiliations:** a Environmental Microbiology Group, University of Dayton Research Institute, Dayton, Ohio, USA; b Fuels and Energy Branch, Aerospace Systems Directorate, Air Force Research Laboratory, Wright-Patterson AFB, Ohio, USA; Louisiana State University

## Abstract

Achromobacter spanius strain 6 is a Gram-negative soil bacterium isolated from a hydrocarbon-degrading microcosm. The draft genome sequence of A. spanius strain 6 is 6.57 Mb with a G+C content of 64.7% and 5,855 protein coding genes. Multiple genes involved in degradation of aromatics are present in this strain.

## Abstract (precis)

Achromobacter spanius strain 6 is a Gram-negative soil bacterium isolated from a hydrocarbon-degrading microcosm. The draft genome sequence of A. spanius strain 6 is 6.57 Mb with a G+C content of 64.7% and 5,855 protein coding genes.

## ANNOUNCEMENT

Achromobacter spanius strain 6 was isolated as a predominant member of the hydrocarbon-degrading bacterial community in a desert soil sample obtained from under a fuel bladder in Kuwait (1). *Achromobacter* spp. have been shown to grow using aromatic compounds (2). The desert soil sample was initially enriched in jet fuel, and the starter enrichment was subjected to 5 successive enrichments with jet fuel. From the fifth enrichment, a pure Achromobacter spanius colony was isolated on Trypticase soy agar (TSA). The prevalence of A. spanius strain 6 in the jet fuel enrichments prompted us to sequence its genome to obtain a better understanding of the metabolic and adaptive pathways in this bacterium. Previously, the whole-genome sequence of P. aeruginosa ATCC 33988 (3) helped researchers to understand the hydrocarbon degradation pathways and fuel-adaptive mechanisms in this bacterium (4–6).

High-quality genomic DNA was isolated from an overnight-grown monoculture of A. spanius strain 6 in lysogeny broth (LB) using the UltraClean Microbial DNA isolation kit, (Mo Bio Laboratories, Carlsbad, CA) and was subjected to DNA library preparation using the SMARTer Apollo library prep system (TaKaRa Bio, Mountain View, CA). The ligated and indexed pre-PCR library was enriched by performing 5 cycles of PCR using the NEBNext high-fidelity 2× PCR master mix. The amplified library was purified for quality control (QC) analysis and sequencing using the Apollo PCR cleanup script and AMPure XP beads (Beckman Coulter, Brea, CA). The purified library was then sequenced using an Illumina HiSeq 1000 sequencer, producing 33,278,050 paired-end reads with a read length of 100 bp. The raw sequence reads were trimmed using Trimmomatic version 0.36 (7) with the following settings: LEADING, threshold quality of 5; TRAILING, threshold quality of 5; SLIDINGWINDOW, average quality of 15 across 4 bp; AVGQUAL, average read quality of 15; and MINLEN, minimum length of 50 bp. The trimmed reads were *de novo* assembled using SPAdes version 3.11.0 (8) with the settings "careful" and "only-assembler." The draft genome assembly comprises 57 scaffolds with an *L*_50_ value of 6 bp and an *N*_50_ value of 425,841 bp. The genome size of A. spanius strain 6 is 6.57 Mb with a G+C content of 64.7% and 5,855 protein coding genes.

Genome annotation was performed using the Prokaryotic Genome Annotation Pipeline (PGAP; https://www.ncbi.nlm.nih.gov/genome/annotation_prok/), and metabolic networks were constructed with Rapid Annotations using Subsystem Technology (RAST) (9). RAST predicted 195, 511, and 211 proteins related to stress response, membrane transport, and metabolism of aromatic compounds, respectively. The 16S rRNA gene sequence of A. spanius strain 6 shares 100% homology with the16S rRNA gene of A. spanius strain DSM 23806. However, the 23S rRNA gene of strain 6 presents four mismatches and a similarity of only 99% with strain DS23806, indicating that A. spanius strain 6 is a different strain. Previous studies have shown that stress response and solvent resistance mechanisms play central roles in the adaptation of bacteria to hydrocarbon fuel (4, 5). The presence of genes related to hydrocarbon degradation, including those involved in catabolism of aromatic compounds, stress response, and membrane transport in the genome of A. spanius strain 6, may explain why this strain is well adapted to jet fuel. The NCBI Prokaryotic Genome Annotation Pipeline predicted multiple genes responsible for aromatic degradation, including naphthalene 1,2-dioxygenase, gentisate 1,2-dioxygenase, catechol 1,2-dioxygenase, and protocatechuate 3,4-dioxygenase. Understanding microbial interactions and bacterial community structure in environments contaminated with hydrocarbons is essential when developing bioremediation strategies (10). The genome sequence of A. spanius strain 6 will help us understand the genetic mechanisms utilized by this organism to thrive in hydrocarbon-rich environments.

### Data availability.

This whole-genome shotgun project has been deposited at DDBJ/ENA/GenBank under the accession number PREU00000000. Raw sequences were deposited in the NCBI SRA database under accession number SRP158285.
